# Postoperative surgical margin results of the phyllodes tumors from a tertiary hospital

**DOI:** 10.1590/1806-9282.20240833

**Published:** 2024-10-07

**Authors:** Fatih Dal, Semiha Battal Havare

**Affiliations:** 1 University of Health Sciences, Turkish Ministry of Health, İstanbul Training and Research Hospital, Department of General Surgery – İstanbul, Turkey.; 2 University of Health Sciences, Turkish Ministry of Health, İstanbul Training and Research Hospital, Department of Medical Pathology – İstanbul, Turkey.

**Keywords:** Breast, Phyllodes tumor, Neoplasm, Recurrence

## Abstract

**OBJECTIVE:**

Phyllodes tumors in the breast are exceptionally uncommon fibroepithelial tumors. In the literature, they are typically categorized as benign phyllodes tumor, borderline phyllodes tumor, and malignant phyllodes tumor. This study aims to assess and present the clinical and surgical outcomes of patients diagnosed with phyllodes tumor.

**METHODS:**

The outcomes of patients aged 18 years and above diagnosed with phyllodes tumor between 2006 and 2023 were retrospectively reviewed. Patients were grouped as benign phyllodes tumor and borderline/malignant phyllodes tumor and compared by clinical and surgical results.

**RESULTS:**

Of all 57 patients with phyllodes tumor, 64.9% (n=37) were benign phyllodes tumor and 35.1% (n=20) were borderline/malignant phyllodes tumor [22.8% (n=13) borderline phyllodes tumor and 12.3% (n=7) malignant phyllodes tumor]. When the patients were divided into two groups as benign phyllodes tumor and borderline/malignant phyllodes tumor and compared, our cumulative (total) recurrence rate was 14.0%, with final surgical margin width between groups [(0<final surgical margin<2 mm vs final surgical margin≥2 mm) (p=0.154)] and recurrence [(8.1% benign phyllodes tumor vs 25.0% borderline/malignant phyllodes tumor) (p=0.080)]; there was no significant difference between our rates.

**CONCLUSION:**

Phyllodes tumors of the breast can be followed up with a narrow negative surgical margin (0 mm<final surgical margin<2 mm). However, after the initial surgery, re-excision is recommended for positive margins, while a wider surgical margin (≥10 mm) is not necessary for excision.

## INTRODUCTION

Phyllodes tumors (PTs) of the breast, first introduced by Muller in 1838, are fibroepithelial breast tumors that originate from neoplastic mesenchymal cells associated with the epithelium of the breast and constitute less than 1% of all primary breast tumors^1–4^. The essential treatment method for the disease is surgery^3,5^. These tumors with a heterogeneous structure are classified by the World Health Organization (WHO) as benign phyllodes tumor (BPT), borderline phyllodes tumor (BrPT), and malignant phyllodes tumor (MPT) based on morphological histopathological parameters (stromal atypia, stromal cellularity, stromal overgrowth, mitotic count, and nature of tumor margin)^2,3,5–8^. Despite this classification with varying grades, the National Comprehensive Cancer Network (NCCN) treatment guidelines recommend excision with a wide surgical margin (SM) (≥10 mm) while neglecting axillary surgery (AS)^9^. However, recent reports indicate no correlation between local recurrence (LR) rates and negative SM width^2,10–13^. This scenario has sparked considerable debate within academic circles regarding the optimal negative or final SM for various classes of PTs post-diagnosis.

In this study, aimed at contributing to the literature, we seek to present the outcomes of surgical treatment in patients diagnosed with PTs of the breast at our clinic between 2005 and 2023.

## METHODS

This study was conducted in accordance with the ethical standards defined by the Institutional Research Committee and the 1964 Helsinki Declaration. Before the study, approval was received from the local ethics committee of the university (decision number and data: 115/12-05-2023).

The study enrolled 57 patients (n=57) aged 18 years and above diagnosed with PT and underwent surgical treatment between 2006 and 2023. Each patient had a minimum follow-up period of 6 months. Demographic, clinicopathological, and treatment outcome data of the patients, whose consent was obtained, were collected from their medical records.

The surgical approach was either local excision (LE) (breast-conserving surgery) or mastectomy. Following LE, positive SMs (presence of tumor cells at the inked tissue edge) were addressed through re-excision or mastectomy to achieve negative SMs. The cases were divided into two groups based on their final negative SM status (0<final SM<2 mm vs. final SM≥2 mm). However, in clinical and radiological axillary lymphadenopathy cases where there was still clinical suspicion following nondiagnostic needle biopsy results, a limited number of patients underwent sentinel lymph node (SLNB) sampling with methylene blue. In cases where methylene blue staining was unsuccessful, axillary lymph node dissection (ALND) was performed. This study assessed surgical samples for stromal atypia, stromal cellularity, stromal overgrowth, mitotic count [per 10 high-power field (HPF)], nature of tumor margin, and necrosis status. The tumors were classified as BPT, BrPT, and MPT based on WHO guidelines. Patients were divided into two groups: BPT and Br/MPT. Patients were compared by their demographic, clinicopathological, and surgical characteristics, follow-up duration, and recurrence.

### Statistical analysis

We used the descriptive statistics of mean, standard deviation, median, minimum, maximum, frequency, and ratio. The Kolmogorov-Smirnov test was used to measure the distribution of the variables. The independent-sample t-test and the Mann-Whitney U test were employed to analyze quantitative independent data. Quantitative independent data were analyzed using the chi-square test, but when the conditions for the chi-square test were not met, we used the Fisher test. The SPSS 28.0 program was used for the analyses.

## RESULTS

Of all 57 patients with PT, 64.9% (n=37) were BPT and 35.1% (n=20) were Br/MPT [22.8% (n=13) BrPT and 12.3% (n=7) MPT]. The average age of the patients was 39.89±13.07 years (range 18.0–63.0 years), and the average tumor size was 6.12±3.87 cm, (range 1.50–20.00 cm). The following surgical procedures were performed for the patients: LE in 77.2%, re-excision or mastectomy due to positive SMs following LE in 15.8% (re-excision in 8.8%, mastectomy in 7%), direct mastectomy in 7%, and AS in 8.8% (SLNB in 7% and ALND in 1.8%). Axillary metastases were not detected in any of the patients. Following surgical treatment, the distribution of final SM in patients was as follows: 56.1% had a margin width greater than 0 but less than 2 mm, and 42.1% had a margin width of 2 mm or greater. After surgical treatment, one patient (1.8%) received both adjuvant chemotherapy (AC) and adjuvant radiotherapy (AR), while the other two patients received only AR. Following treatments, during a follow-up period of 47.15±44.05 months (range 6.0–203.0 months), 14.0% of patients (n=8) experienced recurrence (Table 1).

**Table 1:** Distribution of clinical and demographical data.

		Min–max	Median	Mean±SD-% (n)
Phyllodes tumor	Benign			64.9% (37)
	Borderline/malignant			22.8% (13)/12.3% (7)
Age		18.0–63.0	41.00	39.89±13.07
Age groups	<50			73.7% (42)
	≥50			26.3% (15)
Side	Right			50.9% (29)
	Left			47.4% (27)
	Bilateral			1.8% (1)
Tumor size (cm)		1.50–20.00	5.00	6.12±3.87
Surgery	Local excision			77.2% (44)
	Margin revision after local excision			8.8% (5)
	Mastectomy after local excision			7.0% (4)
	Mastectomy			7.0% (4)
Margin revision	Yes			15.8% (9)
	No			84.2% (48)
Final surgical margin (mm)	0 mm<surgical margin<2 mm			56.1% (32)
	Surgical margin≥2 mm			42.1% (24)
Axillary surgery	Axillary surgery negative			91.2% (52)
	Sentinel lymph node biopsy			7.0% (4)
	Axillary lymph node dissection			1.8% (1)
Adjuvant chemotherapy	+			1.8% (1)
	-			98.2% (56)
Adjuvant radiotherapy	+			5.3% (3)
	-			94.7% (54)
Mitotic count (×10 High-power field)	≤4			66.7% (38)
	5–9			21.1% (12)
	≥10			12.3% (7)
Stromal atypia	None or mild			47.4% (27)
	Moderate or marked			43.9% (25)
Stromal cellularity	Yes (Moderate or marked)			91.2% (52)
	No (None or mild)			1.8 (1)
Stromal overgrowth	+			38.6% (22)
	-			43.9% (25)
Necrosis	+			7.0% (4)
	-			93.0% (53)
Recurrence	+			14.0% (8)
	-			86.0% (49)
Follow-up duration (month)		6.00–203.00	30.00	47.15±44.05

When the patients were divided into two groups, namely BPT and Br/MPT, and compared, the rate of undergoing surgical LE in BPT was significantly higher (p=0.002), and this group consisted of significantly younger patients (p=0.017). Histopathologically, the mitotic count (10× HPF) (p<0.001) and the rate of stromal atypia were significantly higher in Br/MPT (p<0.001). The overall recurrence rate among patients was 14.0% (n=8), with LR observed in seven patients and aggressive progression with lung metastasis in one patient who received both AC and AR. The other two patients receiving AR after surgical treatment were in remission. Although there was no significant difference in recurrence rates between the two groups during follow-up after treatment [BPT: 8.1% (n=3) vs. Br/MPT: 25.0% (n=5)], the Br/MPT group had significantly lower rates of additional SM revisions (p=0.031), axillary surgical interventions (p=0.015), and radiotherapy (p=0.039) (Table 2).

**Table 2:** Comparison of the groups.

		Benign phyllodes tumor		Borderline/malignant phyllodes tumors		p
		Median	Mean±SD-% (n)	Median	Mean±SD-% (n)	
Age		39.00	36.97±12.28	47.50	45.30±13.08	**0.017** ^m^
Age groups	<50		83.8% (31)		55.0% (11)	**0.019** ^x2^
	≥50		16.2% (6)		45.0% (9)	
Side	Right		48.6% (18)		55.0% (11)	0.712^x2^
	Left		48.6% (18)		45.0% (9)	
	Bilateral		2.7% (1)		0.0% (0)	
Tumor size (cm)		4.00	5.40±3.42	6.65	7.45±4.39	0.056^t^
Surgery	Local excision		91.9% (34)		50.0% (10)	**0.002** ^x2^
	Margin revision after local excision		5.4% (2)		15.0% (3)	
	Mastectomy after local excision		2.7% (1)		15.0% (3)	
	Mastectomy		0.0% (0)		20.0% (4)	
Margin revision	Yes		8.1% (3)		30.0% (6)	**0.031** ^x2^
	No		91.9% (34)		70.0% (14)	
Final surgical margin (mm)	0 mm<surgical margin<2 mm		64.9% (24)		42.1% (8)	0.154^x2^
	Surgical margin≥2 mm		35.1% (13)		57.9% (11)	
Axillary surgery	Axillary surgery negative		97.3% (36)		80.0% (16)	**0.015** ^x2^
	Sentinel lymph node biopsy		0.0% (0)		20.0% (4)	
	Axillary lymph node dissection		2.7% (1)		0.0% (0)	
Adjuvant chemotherapy	+		0.0% (0)		5.0% (1)	0.351^x2^
	-		100.0% (37)		95.0% (19)	
Adjuvant radiotherapy	+		0.0% (0)		15.0% (3)	**0.039** ^x2^
	-		100.0% (37)		85.0% (17)	
Mitotic count (×10 High-power field—HPF)	≤4		91.9% (34)		20.0% (4)	**<0.001** ^x2^
	5–9		8.1% (3)		45.0% (9)	
	≥10		0.0% (0)		35.0% (7)	
Stromal atypia	None or mild		71.9% (23)		20.0% (4)	**<0.001** ^x2^
	Moderate or marked		28.1% (9)		80.0% (16)	
Stromal cellularity	Yes (Moderate or marked)		97.0% (32)		100.0% (20)	1.000^x2^
	No (None or mild)		3.0% (1)		0.0% (0)	
Stromal overgrowth	+		37.0% (10)		60.0% (12)	0.148^x2^
	-		63.0% (17)		40.0% (8)	
Necrosis	+		2.7% (1)		15.0% (3)	0.119^x2^
	-		97.3% (36)		85.0% (17)	
Recurrence	+		8.1% (3)		25.0% (5)	0.080^x2^
	-		91.9% (34)		75.0% (15)	
Follow-up duration (month)		32.00	53.51±48.76	22.50	35.38±31.45	0.139^t^

^t^t-test/^m^Mann-Whitney U test/^x2^ chi-square (Fisher test). Statistically significant values are shown in bold.

## DISCUSSION

Given the limited data, it is not possible to discuss conclusively the exact cause of PTs. However, reports in the literature suggest associations with increased growth factors secondary to hyperestrogenism, genetic mutations, Li-Fraumeni syndrome, androgen insensitivity syndrome, and gonadal malignancies^3–5,10,14^. It is frequently mistaken for breast fibroadenomas in the differential diagnosis. Radiological methods aid in the diagnosis and guide for selecting the appropriate surgical management after diagnosis, supporting optimal diagnostic accuracy. Although radiologically breast fibroepithelial lesions larger than 3 cm are likely to be PT, the definitive diagnosis of the disease is made after histopathological evaluation of the tumor^5,7,8,15,16^.

Patients typically present to the clinic with rapidly growing painless unilateral, bilateral, and multifocal masses and/or lumps in the breast. The sizes of these masses range from 0.5 to 30 cm, with average dimensions of 5–7.2 cm^2–4,11,14^. In the literature, tumors larger than 5 cm are associated with an increased risk of LR^11,12,17^. To assess its clinical behavior and prognosis, WHO categorizes PTs into different grades, namely BPT, BrPT, and MPT, based on histopathological factors like stromal atypia, stromal cellularity, stromal overgrowth, mitotic count, and the nature of the tumor margin^2,3,5–8^. Tumors are distributed in the literature according to their grades as follows: approximately 64–72.7% are BPT, 11.5–18% are BrPT, and 8.9–18% are MPT^5,18^. In a study by Rosenberger et al. with 550 cases, the grade of PT, tumor size, stromal atypia, and stromal overgrowth were significantly higher in patients with LR (p<0.05). However, in the same study, mitotic activity (threshold value=10) was not significant (p>0.05)^10^. In our study, the distribution of tumor grades was similar to the literature. The rate of stromal atypia in Br/MPT was significantly high (p<0.001), supporting the literature. However, although tumor size, stromal overgrowth, and necrosis rates were high in Br/MPT in line with the literature, there was no significant difference between the groups (p>0.05). Additionally, due to the study design, the rate of mitotic activity (threshold value=4) was significantly higher in Br/MPT (p<0.001).

The disease is most commonly observed in women during the third and fifth decades of life^2,3,5,10–12,19^. It is reported in the literature that patients with Br/MPT are younger^11,20^. Moreover, according to a study by Wei et al. with 192 cases, younger age, larger tumor size, higher tumor grade, and positive margins were associated with LR^21^. However, in a study by Yılmaz et al. investigating 26 cases, Br/MPT patients were older^5^. In our study, the patients with Br/MPT were similarly older. Although our results are consistent with the findings of Yılmaz et al., the limited number of cases in our study may have led to this different result.

It is recommended to opt for LE instead of AS and mastectomy for these tumors, which have a heterogeneous structure. Additionally, after surgical treatment, patients may have recurrences, mostly localized, at rates ranging from 9.4 to 21%. Systemic recurrences are often associated with Br/MPT and are rare^2,3,5,10,22^. Due to the risk of recurrence, NCCN guidelines recommend a negative SM of at least 10 mm for these tumors^9^. However, the excision of these tumors with wide SMs (≥10 mm) may lead to poor cosmetic outcomes. Tukenmez et al. reported the necessity of ensuring at least a 10-mm SM for Br/MPT^17^. However, in the study by Lim et al., they reported an increased risk of recurrence in Br/MPT when the SM was positive or ≤1 mm^13^. Similarly, in a study by Boland et al., where the authors evaluated the SM status (SM positive, 0<SM≤2 mm, SM>2 mm), they found that tumors with positive margins and MPTs had a higher risk of recurrence^19^. Additionally, in a study comparing BPT and BrPT based on the SM status (SM positive, 0<SM<2 mm, SM≥2 mm), Moldoveanu et al. reported an association between initially positive SMs and increased recurrence^22,23^. As seen in the literature, while a negative SM (no tumor cells touching ink or no ink tumor<10 mm) is deemed sufficient for BPT, there is a prevailing consensus that in Br/MPT, LE with narrower margins below 10 mm and, in the case of positive SMs, a staged, more extensive resection (re-excision) is advisable^3,10,12,17,19^. According to our results, the mastectomy and SM revision rates were significantly high (p=0.031) in Br/MPT. Similar to the literature, our cumulative recurrence rates were 14.0%, with no significant difference between groups in terms of the final SM width [(0<final SM<2 mm vs. final SM≥2 mm) (p=0.154)] and recurrence rates [(8.1% BPT vs. 25.0% Br/MPT) (p=0.080)]. According to our results, PT negative can be followed with a narrow SM (0 mm<final SM<2 mm).

Lymphatic metastasis is not expected in PTs originating from the mesenchymal-derived stromal structures of the breast. Therefore, routine AS is not recommended. However, AS is indicated if the surgeon has concerns about simultaneous invasive adenocarcinoma if the patient has palpable axillary lymph nodes, and/or if there are histopathologically confirmed positive axillary lymph nodes. With increased knowledge about the clinical behavior of the disease in the literature, our rates of AS have decreased from around 25 to 2%. The axillary metastasis rate currently varies between 0 and 5%^2,5,10,24^. In our study, AS was performed on 8.8% of the patients (n=5), and no axillary metastasis was detected in any of the patients.

Our knowledge about the role of adjuvant treatments such as AC and adjuvant hormone therapy in PT post-surgery is limited, and there is no clear evidence or treatment protocols for reducing LR^4,5,8^. Although it has been reported in the literature that AR after surgery provides local control and reduces recurrence in Br/MPT, it is still debated due to the increased risk of secondary malignancy associated with genetic mutations after AR^3,4,10,11,24,25^. However, NCCN recommends wide excision and AR without axillary staging in the case of LR (level of evidence 2B), and systemic metastasis cases are suggested to be managed similarly to soft tissue sarcoma^9^. In our study, although the rate of AR was significantly high in Br/MPT, the limited sample size (n=3) emphasizes the need for studies with higher levels of evidence regarding adjuvant treatments.

As mentioned earlier, the study's retrospective nature and the disease's rarity limit the sample size investigated in the present study.

## CONCLUSION

PTs of the breast can be followed up with a narrow negative SM (0 mm<final SM<2 mm). However, after the initial surgery, re-excision is recommended for positive margins, while a wider SM (≥10 mm) is not necessary for excision. As for adjuvant treatments, there is a need for further extensive prospective studies.

## ETHICAL APPROVAL

This study was conducted in accordance with the ethical standards defined by the Institutional Research Committee and the 1964 Helsinki Declaration. Before the study, approval was received from the local ethics committee of the University of Health Sciences Istanbul Training and Research Hospital (decision number and data: 115/12-05-2023).
